# The Case Report of a 97-Year-Old Patient With Chronic Anemia and Hemoglobin Value of 1.7 g/dl and Review of the Literature

**DOI:** 10.7759/cureus.24330

**Published:** 2022-04-21

**Authors:** Andreas Kyvetos, Stefani Panayiotou, Panagiota Voukelatou, Ioannis Vrettos, Georgios Boulmetis

**Affiliations:** 1 2nd Department of Internal Medicine, General and Oncology Hospital of Kifissia “Agioi Anargyroi”, Athens, GRC

**Keywords:** adaptive, deficiency, iron, anemia, severe

## Abstract

Although hemoglobin levels beneath 6.5 g/dl are considered to be life-threatening and the patients theoretically suffer from a cluster of symptoms, few cases of patients who seek medical assistance when their hemoglobin levels had fallen beneath 3 g/dl have been reported in the literature. Here, we describe the case of a 97-year-old patient who was transferred to the emergency department with dyspnea and the initial screening tests showed a hemoglobin level of 1.7 g/dl, due to iron deficiency. The patient was hemodynamically stable, and no ischemic lesions were seen on the electrocardiogram. His dyspnea was due to a lower respiratory tract infection and bilateral pleural effusion. He was bedridden for two years. His absence of physical activity in combination with the slow onset of anemia and the absence of severe underlying pulmonary and cardiovascular diseases could hide the symptoms until additional stressful events, such as the respiratory tract infection and the deterioration of heart function, occurred. So, we must keep in mind that in elderly patients with reduced physical activity and without severe pulmonary and cardiovascular comorbidities, the symptoms of severe anemia may go unnoticed until hemoglobin reaches life-threatening levels.

## Introduction

The symptoms of anemia like dyspnea, tachypnea, palpitations, cold skin, heart failure, cognitive dysfunction, etc. depend, inter alia, on the severity of anemia, the rapidity of its onset, the age, and the physiological status of the patient. Various adaptive mechanisms are mobilized by the human body in order to counterbalance the effects of anemia, although almost every organ system is finally affected [1]. The most significant adaptive responses involve the cardiovascular system. These adaptive responses include increased cardiac output and redistribution of blood flow toward the heart and central nervous system and away from the splanchnic vascular beds [2].

In cases of chronic anemia, it has been postulated that the hyperkinetic response in patients at rest, occurs only when hemoglobin level falls beneath 7 g/dl [3]. Moreover, in hemoglobin levels beneath 7.5 g/dl, 100 ml of arterial blood contains 10 ml of oxygen instead of 20 ml in healthy subjects [1]. Nevertheless, certain symptoms or complications experienced by a patient cannot be determined with the accuracy of hemoglobin levels alone [4]. Heart, lung, and cerebrovascular diseases, as well as age, may limit the adaptive responses to anemia [2]. On the other hand, in young and otherwise healthy persons, chronic anemia may remain unnoticed until hemoglobin level falls beneath a critical level or episodes of exertional stress occur [1].

According to several grading systems for anemia, hemoglobin levels beneath 6.5 g/dl are considered life-threatening [5], and the patient theoretically suffers from a cluster of symptoms. However, a few cases of patients have been reported in the literature [6-12] who seek medical assistance when their hemoglobin levels had fallen beneath 3 g/dl and therefore their hematocrit levels were beneath 10%.

In this report, we describe the case of a “too hard to die” 97-year-old patient who was transferred to the emergency department with dyspnea and the initial screening tests showed a hemoglobin level of 1.7 g/dl, and we review and discuss the reported cases of other “too hard to die” patients with hemoglobin concentrations beneath 3.0 g/dl due to chronic anemia. This paper is an extended version of a work presented at the 24ο Panhellenic Congress of Internal Medicine, Athens, Greece, on 3-6 November 2021.

## Case presentation

A 97-year-old patient was admitted to the emergency department due to shortness of breath for one week, without other symptoms. According to his medical history, the patient suffered from anemia, treated with 247.25 mg ferrous sulfate, 5 mg folic acid daily, and hypothyroidism treated with levothyroxine sodium 75 mcg. His relatives were aware of his anemia but they preferred to treat it with per-os medication without investigating the cause of the anemia. They reported that the patient’s stools for several days were tarry but they believed it to be due to the iron he was receiving. Six months ago, he was diagnosed with pulmonary embolism which was effectively managed with apixaban 110 mg twice daily and two years ago he had an ischemic stroke that left him bedridden.

**Figure 1:**
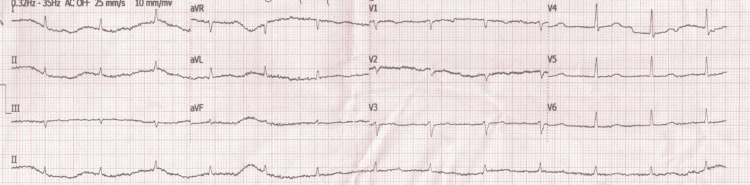
The electrocardiogram of the patient showing the absence of ischemic lesions

At the time of presentation, he appeared pale and lethargic (Glasgow Coma Scale 7/15) with tachypnea (30 breaths/min). Bilateral crepitations and rhonchi were present in lung auscultation. There was a systolic murmur throughout the precordium. A digital rectal examination was performed and it was negative for melena. His blood pressure was 120/60 mmHg with a regular pulse rate of 90 beats/min. Other findings from the physical examination were unremarkable. The laboratory test revealed hypochromic microcytic anemia, with hemoglobin value of 1.7 g/dl (normal range: 12-16 g/dl), hematocrit 6.9% (normal range: 37-48%), red blood cell count of 1.53 Μ/ml (normal range: 4.5-6.3 Μ/ml), mean corpuscular volume of 75 fl (normal range: 80-96 fl), and mean corpuscular hemoglobin of 23.8 pg (normal range: 27-34 pg). The complete blood count was examined twice in order to avoid a laboratory error in measurement. Other laboratory results showed: white blood cells 6.47 K/μl (normal range: 4.0-11.0 Κ/μl), platelets 121 Κ/μl (normal range: 150-400 Κ/μl), C-reactive protein 3.56 mg/dl (normal range: <0.5 mg/dl), creatinine 1.7 mg/dl (normal range: 0.6-1.4 mg/dl), urea 135 mg/dl (normal range: 10-50 mg/dl), total bilirubin 0.7 mg/dl (normal range: 0.2-1 mg/dl), ferritin 20 ng/ml (normal range: 11-204 ng/ml), folicid acid >40 ng/ml (normal range: 3.1-20 ng/ml), cobalamin 700 pg/ml (normal range: 187-883 pg/ml), erythrocyte sedimentation rate 10 mm/h (normal range: 2-20 mm/h), direct Coombs test negative, a normal urinalysis test, high sensitivity troponin I (HS-TnI) 34 pg/ml (normal range: <11.6 pg/ml), and brain natriuretic peptide (BNP) 804.30 pg/ml (normal range: <100 pg/ml). No ischemic lesions were seen on the electrocardiogram (Figure 1) and the second sample of HS-TnI was within the normal range. Hematological consultation was requested and the blood smear revealed normal platelets count of 199 Κ/μl and microcytic red blood cells with hypochromia. Erythropoietin levels and total iron-binding capacity were not performed due to the lack of these laboratory tests in our hospital.

**Figure 2:**
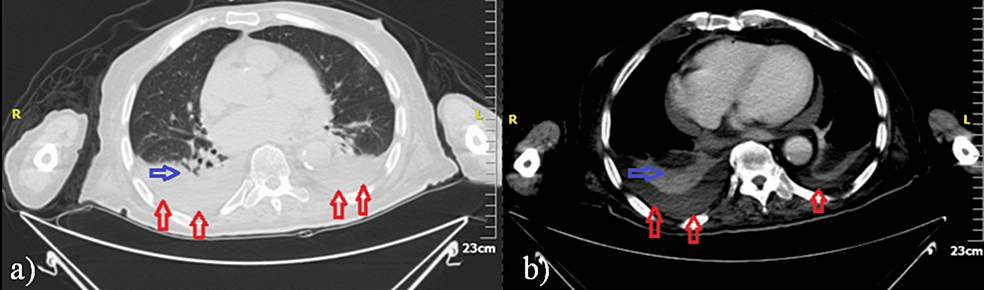
Chest CT scan images a) Lung window b) Mediastinal window It revealed a lower respiratory tract infection (blue arrows) and bilateral pleural effusion (red arrows).

Cardiac echocardiography was performed that showed signs of heart failure with preserved ejection fraction and grade I diastolic dysfunction. A chest CT scan revealed a lower respiratory tract infection and a bilateral pleural effusion (Figure 2) compatible with his heart failure. An abdominal CT scan was performed that did not reveal any pathology.

The patient was treated with furosemide, ceftriaxone 2 g once daily and clindamycin 600 mg three times daily. A total of 5 units of packed RBCs were transfused resulting in a hemoglobin value of 8.7 g/dl. The renal function at the admission was impaired but gradually it was improved and the discharge creatinine was 0.8 mg/dl. Gastroscopy and colonoscopy were scheduled but his relatives refused any further investigation to identify the cause of anemia. Although the investigation for the cause of anemia was inadequate, the history of tarry stools in a patient receiving anticoagulant, in combination with a ferritin value of 20 ng/ml made us assume that the main cause of his anemia was chronic bleeding from the gastrointestinal tract. So, he received 1 g of intravenous iron. The patient had a remarkable recovery to his preadmission state and he was discharged home 11 days after.

## Discussion

Our patient had one of the lowest hemoglobin levels that have ever been reported. To our knowledge, the lowest hemoglobin concentration that has ever been reported in a non-trauma patient is 1.2 g/dl (hematocrit of 3.0%) due to paroxysmal nocturnal hemoglobinuria. The 21-year-old male patient was presented with profound weakness and abdominal pain, although hemodynamically stable [6]. The second-lowest reported hemoglobin was 1.3 g/dl (hematocrit of 4.7%) and it was due to iron deficiency, because of chronic uterine bleeding [7]. The patient was a 44-year-old woman with weakness and dyspnea at rest. She had regular pulses, normal arterial pressure, and 25 breaths per minute. She had severe lactic acidosis and inverted T waves in leads III, aVF in her ECG.

Likewise, an extremely low hemoglobin level, in a patient with chronic anemia, has been reported by Jost et al. They presented a 29-year-old woman with celiac disease, bulimia nervosa, and iron-deficiency anemia. She had a hemoglobin level of 1.7 g/dl. She was malnourished and she reported exhaustion, fatigue, and abdominal pain but no critical symptoms [8]. On the other hand, a 76-year-old woman had critical symptoms with hemoglobin 2.4 g/dl due to gastric adenocarcinoma. She was hemodynamically unstable and reported increased fatigue, reduced activity, intermittent nausea or vomiting, and melena. Clarke and Weston-Smith reported a case of folate deficiency in a 50-year-old woman with hemoglobin of 2.6 g/dl. They reported that the patient was stable, alert, and keen to avoid admission. She had a soft ejection systolic murmur and her ECG was in normal sinus rhythm of 90 beats per minute with ST-segment depression in lateral leads [10].

Another case that was presented by Reibke et al. was a 32-year-old male with hemoglobin 2.9 g/dl due to B12 deficiency and minor beta-thalassemia [11]. Finally, extremely low hemoglobin levels have been reported by Bhatia et al., who performed coronary hemodynamic studies on 14 patients with chronic anemia. They reported three patients with hemoglobin levels ≤3.0 mg/dl; they had hemoglobin levels of 1.6 mg/dl, 2.4 mg/dl, and 3.0 mg/dl, and all of them were between 23 to 25 years old. Two of them had iron-deficiency anemia due to ankylostomiasis and the third had B12 deficiency [12].

All of these patients had no underlying pulmonary and cardiovascular diseases and they developed gradually severe anemia. This gradual onset allowed the compensatory mechanisms to take place and so, the patients arrived at the emergency departments with hemoglobin levels much lower than that considered to be life-threatening. The difference with our patients is that almost all, with one exception [9], were less than 50 years old and so, theoretically, they had satisfactory adaptive responses to anemia. The exception [9] was referred to as a 76-year-old woman with gastric adenocarcinoma that arrived at the emergency department with hypotension and tachycardia. This woman had symptoms compatible with anemia for several weeks.

In contrast, our patient was hemodynamically stable, and he had no profound symptoms until the time dyspnea was developed due to both the lower respiratory tract infection and also the deterioration of cardiac function which was triggered probably both by anemia and the respiratory tract infection. What hid the patient’s symptoms from his relatives, according to our opinion, was the non-existent, even the minimal, physical activity of this elderly. His absence of physical activity in combination with the slow onset of anemia and the absence of severe underlying pulmonary and cardiovascular diseases could hide the symptoms until the time of additional stressful events, such as the respiratory tract infection and the deterioration of heart function, occurred.

## Conclusions

Symptomatic anemia is a common finding in elderly patients. Whereas, in elderly patients with reduced physical activity and without severe pulmonary and cardiovascular comorbidities, the symptoms of severe anemia may go unnoticed until hemoglobin reaches life-threatening levels. Careful surveillance of these patients is mandatory with regular clinical examination and laboratory blood tests, especially for those who are receiving anticoagulant therapy.
